# The impact of AI assimilation on firm performance in small and medium-sized enterprises: A moderated multi-mediation model

**DOI:** 10.1016/j.heliyon.2024.e29580

**Published:** 2024-04-14

**Authors:** Mohamad Deeb Abdul Wahab, Mehrshad Radmehr

**Affiliations:** Economics and Administrative science, Department of Business Administration, Cyprus International University, Nicosia, Northern Cyprus, Via Merson 10, Turkey

**Keywords:** Artificial intelligence, Firm performance, SMEs, Lebanon

## Abstract

Artificial intelligence (AI) and other advanced technologies are increasingly recognized as essential catalysts for enhancing productivity due to their capability to transform nearly all operations within and outside firms. However, the empirical research on how AI assimilation may promote firm-level outcomes such as absorptive capacity (AC), customer agility (CA), and firm performance (FP) is still in its infancy. Drawing from the dynamic capability view and using 417 valid responses collected through cross-sectional methods from small and medium-sized enterprises (SMEs) in Lebanon, this study examines the effect of AI assimilation on firm performance. The mediating roles of AC and CA were investigated. The moderating role of organizational agility (OA) was also explored. The findings support the hypothesized assumptions that continual advancement of technology evolves the industrial organizations' performance with CA and AC as parallel mediators, partially mediating the link between AI assimilation and FP and OA as a moderator, moderating the positive relationship between AI and CA and between AI and FP. The findings provide crucial insights for practitioners and advance the dynamic capability view framework. They provide compelling evidence that enriches the understanding of AI assimilation, demonstrating its positive impact on critical organizational outcomes and yielding performance benefits for SMEs.

## 1 Introduction

The modern era requires modern solutions, and existing businesses are deploying technologies to meet customers’ expectations [1]. Customers want quick responses, which necessitates the use of fast-moving systems and responsiveness. From this standpoint, firms are introducing AI technologies to establish a CA system that can quickly respond to an ever-changing business environment. Through such technologies, firms can improve problem-solving abilities, boost productivity and efficiency in customer service and other fields [2], and improve their performance and productivity [[3], [4], [5], [6], [7], [8], [9]].

The use of AI technology to assist organizations in attaining and maintaining a competitive edge relies on the complete assimilation of such technology throughout business processes [10]. This is due to IT assimilation's crucial role in enabling end-to-end business operations to develop and maintain business value [10,11]. Existing literature has acknowledged that it can be counted on to enhance organizational performance [12] and foster supply chain agility and business value [13]. However, most of the existing literature focuses on only two facets of assimilation: the crucial factors of assimilation [10,14] and IT-based outcomes [10,15].

The assimilation of AI is an emerging topic that requires additional research. Up to this point, not much has been written concerning AI assimilation, even regarding high-level strategic and operational performance, either in practice [16] or in theory [[17], [18], [19]]. However, an increasing number of studies has already recognized the potential of AI's high business value [[20], [21], [22]], particularly for enhanced productivity [23,24] and innovative performance at the firm level [25,26]. In addition [27], emphasized the role of personal cultural orientations in fostering AI acceptance among SMEs. Despite this, it has been noted that most companies we engage directly or interact with have not yet adopted AI [28].

Additionally [29], suggested that more than the application of AI is required to improve firm performance. In a similar vein [30], particularly pointed out that AI will be a crucial determinant of how SMEs evolve. Thus, research must comprehend AI's fundamental capabilities and how it will be integrated into SMEs to promote its positive impact on the business sector's future [31]. Besides [32], pointed out contradictory results in the previous literature on the relationship between SMEs' dynamic capabilities and their performance. Accordingly, the link between AI assimilation and FP^1^ can be markedly significant for SMEs but requires more investigation. Hence, this is one of the initial efforts to fill the void aforementioned in the current literature, particularly for SMEs, from an emerging economy perspective [39]. showed that AI in marketing significantly affects financial performance, customer performance, internal business process performance, and learning and growth performance in the case of SMEs in Ghana [40]. revealed the mediating role of accounting automation on AI adoption in the SME context [41]. highlighted that the nexus between AI and competitiveness is positively significant in SMEs. Focusing on 326 SMEs in the U.S. [42], highlighted the significant positive nexus between (AI)-driven business model innovation and both technological and strategic enablers for carbon-neutral businesses [43]. uncovered a diverging pattern of the effect of AI on productivity and innovation growth in SMEs [44]. revealed a significant effect of AI on SMEs' operational and economic performance [45]. indicated that adopting the Industrial Internet of Things in SMEs will upsurge organizational performance [46]. found that smart technology has a key impact on the performance of SMEs in Bahrain.

The emerging literature has highlighted that firms can leverage AI assimilation to improve performance [47,48]. Some studies also showed that firms can improve performance through supply chain orientation and agility [49,50], however, how AI assimilation influences FP remains to be investigated, mainly since the limitation in data availability, as a constraint, has made it difficult to assess this relationship comprehensively [24]. Further, firms also need to leverage the voice of their customers to acquire information regarding the market and recognize opportunities for competitive actions [51]. Fulfilling these needs will help companies develop customer agility, which relates to a firm's capacity to detect and quickly respond to customer-oriented opportunities for innovative and competitive action [52]. Previous research has acknowledged that customer analytics is essential in the link between IT capabilities and FP [53,54]. Based on this, CA may mediate the link between AI assimilation and FP.

While the recent research of [47] has enhanced our understanding of AI assimilation, the literature still lacks a comprehensive understanding of its performance-related outcomes. For example, how AI assimilation promotes AC is still unclear. Similarly [55], indicated that the current literature still needs to gain knowledge about the contingent role of AC on companies’ performance. AC relates to a collection of established procedures and practices organizations use to obtain, assimilate, modify, and utilize knowledge to generate dynamic organizational capability [56]. Though [47] reported that AI assimilation has performance implications, the mechanisms through which AI assimilation leads to FP are not clearly understood from the extant literature. Hence, the current research responds to the research call by Ref. [47] by examining CA and AC as mediators in the AI-FP link.

Furthermore, our understanding of under what conditions AI assimilation influences absorptive capacity, customer agility, and FP still needs to be investigated further in the current literature. Environmental instability and increased ambiguity in the world markets, combined with accelerated technological advancement, have driven firms to recalibrate their capability to respond promptly and malleably adapt [57]. Organizational agility, which relates to the ability to detect and respond quickly to market opportunities at the corporate level [51], could offer a potential lens to account for the influence of AI assimilation on firm performance. Yet, research still has not considered organizational agility's potential moderating role.

**Fig. 1:**
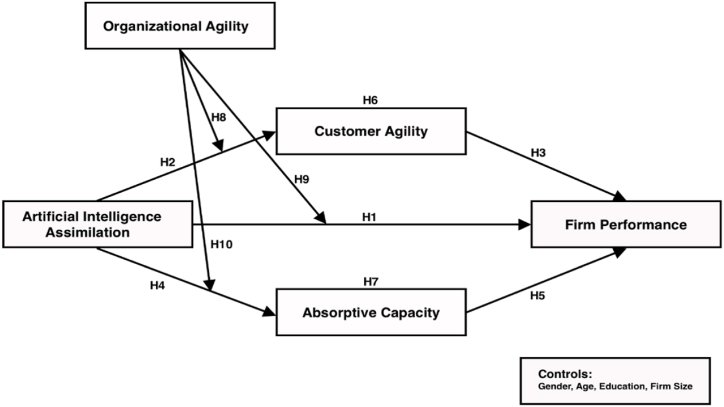
Conceptual framework.

This study fills the gaps mentioned in the current literature by drawing on the Dynamic Capability View framework. Specifically, its primary purpose is to provide a more comprehensive understanding of the relationship between AI assimilation and firm performance. We developed a moderated parallel multi-mediation model to investigate the associated mechanisms and determine whether the relationships, illustrated in Fig. 1, are contingent on organizational agility. Hence, we expand the existing body of knowledge in several ways.

First, we aim to extend the emerging research on AI assimilation and its performance-related outcomes. Second, given the crucial role of AI assimilation for FP in academics [29] and practice [58], research and practice need to examine the underlying mechanisms in such a relationship. This study aims to demonstrate that AC and CA are mechanisms through which AI assimilation improves FP in SMEs from an emerging economy perspective. Third, while the initial effort by Ref. [47] sheds some light on the relationship mentioned above, there is limited empirical research concerning the relationship, and little is known about whether certain constructs would moderate this relationship. Specifically, much remains undisclosed under what particular conditions AI assimilation drives firm performance. Besides, although the initial effort of [47] focused on supply chain samples in a developed economy, SMEs should receive more academic attention, given their importance to the national economy [59]. Therefore, we aim to provide new insights into the contingency role of OA in the AI assimilation-FP relationship.

## 2 Literature review

### 2.1 Theoretical background: dynamic capabilities view

The Dynamic Capabilities View (DCV) theory has received significant scholarly attention to better comprehend the significance of an organization's strategic capabilities and their influence on firm-level competitive performance [60]. In this area of study, scholars asserted that the dynamic capabilities theory may assist in explaining the disparities in business performance within a particular sector [12] while offering enterprises the optimal chance to develop and optimize their resources [61]. The DCV strongly emphasizes maintaining and growing the superior operational capabilities required to carry out and synchronize operational procedures [62]. It is founded on the notion that dynamic capabilities can change a firm's current positions, resources, and operational capabilities, resulting in new positions and avenues for the optimal utilization of its strategic assets [62]. In addition, the DCV elucidates the integration and reconfiguration of internal and external competencies in developing innovations to adapt to new business environments [63]. Based on this, it is unsurprising that prior studies have utilized DCV to comprehend IT-associated capabilities and organization-level outcomes (e.g., Refs. [[63], [64], [65], [66]]). Remarkably [67,68], found that the dynamic capabilities positively impact FP both before and during COVID-19. However, the theory has been more extensively studied in developed countries even though developing markets have distinct capabilities and resources regarding the socio-cultural and economic environment [69]. Thus, we consider this theoretical framework appropriate for our study to develop our theoretical model from a developing economy perspective.

### 2.2 Artificial intelligence assimilation

While the earliest articles on AI date back to the 1940s [70], AI has only recently earned much interest among scholars and practitioners. This is primarily due to the abundance of "big data," which facilitated the onset of what is referred to as ''data-based AI," as well as the emergence of cloud computing, the machines' computing powers, and deep learning's advancements [71]. AI's ability to reinvent nearly every facet of business management in favor of greater productivity and long-term competitive advantage may account for firms' increasing interest in this technology [11]. For instance, AI may be utilized to enhance customer service experience [72,73] leading to improved organizational performance, such as non-financial and financial performance [5,74] and strengthening environmental performance [75,76]. [13] argued that the use of AI allows supply chains to dynamically respond to unstable environments, and lessen possibly costly decision-making for SMEs.

According to Ref. [77], AI assimilation is described as the integration of technology throughout an organization's dimensions of work and project procedures, as well as the routinization of technologies in those activities. Because of its potential significance in boosting operational efficiency and market agility for the continued existence of business enterprises, it has become a key research topic [66]. While IT assimilation is a crucial sign of successful IT deployment, IT-empowered business value creation, and improved FP [78], it also reveals if a firm's investment in IT infrastructure has been successful [12] or has resulted in the anticipated increase in firm's effectiveness and efficiency [79]. The assimilation of IT remains the crucial barometer to measure the effect of investments in the execution of novel strategic technologies, such as their benefit to IT-empowered digital procedures [80] and enhanced strategic and operational rewards [12]. Additionally, IT assimilation is a firm process confirming that IT capability aligns with the organization's strategic decisions on cross-firm partnerships (e.g., managing customer relationships) [11].

IT assimilation is considered by some scholars to be a collection of unique, essential, and imperfectly imitable competencies that enterprises mostly require to obtain a competitive edge in a dynamic and turbulent environment [12]. Given the uncertain and dynamic climate in which SMEs operate, we propose that the integration and routinization of technology throughout the entire operational activities of SMEs should develop their capabilities to support their performance in the best possible manner [45,75,81].

### 2.3 Organizational agility

Agility is best understood as an organization's asset, a collection of organizational practices and procedures that yield a specific result [82]. Therefore, organizations must develop agility [53]. In addition, manufacturing firms are urged to comprehend the impact of agility and create a robust framework to enhance their overall performance [48,83]. Authors have characterized agility in several ways, such as a process [51] and a convergence of IT and strategic agility [84]. It was also revealed that OA could be enhanced by various factors, one of which is fostering workforce agility [85]. It is reported that OA, as an organization's capacity, strengthens firms to respond promptly to changes in the market [86]. [87] showed that both routine and innovative use of chatbots were positively linked with internal and external agility [88]. shed light on the considerable role of OA in carrying out digital transformation inside organizations.

Furthermore [51], emphasized that OA is a firm's capacity to recognize various opportunities in a deficient market and take required precautions to promptly deal with the circumstance and seize such opportunities. Agile firms possess the flexibility to rapidly seek innovative ways of doing things, proactively analyze and adapt to events, and take advantage of opportunities [89]. [90] suggested that OA allows organizations to leverage their diverse assets and adapt to industry developments, which are often unpredictable and necessitate speed and proactivity in operational procedures if the organization wishes to capture opportunities and become an industry leader. Furthermore, it is increasingly seen as an invaluable asset that firms utilize to obtain information required to improve and manage decision-making [91] and acquire a competitive edge [[92], [93], [94]]. Recently, researchers contended that OA research is majorly associated with firms' responses to ancillary processes. Hence, to fully comprehend the inherent processes, it is necessary to offer a more suitable explanation [95].

### 2.4 Customer agility

CA is described as the extent to which an organization can recognize and respond rapidly to customer-based opportunities for innovative and competitive actions [53]. The definition encompasses the previously established fundamental components of agility, such as capability, sensing, response, and speed [53]. The study further argued that CA is a crucial capability in a hypercompetitive environment. It allows organizations to not only quickly discover the preferences and behaviors of their customers by observing data related to the customers but also monitor prospective customers in real-time, thereby enhancing customers' interaction and experience [89], and customized retrieval of information [96]. CA enables enterprises to leverage customer feedback to obtain information regarding the market and analyze competitive initiative opportunities, facilitating the firm's survival and growth [96,97]. Per the results of the Importance-Performance Map Analysis (IPMA), CA is one of the most critical dimensions of OA and should be carefully controlled within organizations [98]. In this research, we follow the perspective of [92] regarding OA and propose that OA and CA may act as an organizational condition, which can offer a more suitable explanation for better comprehending the link between AI assimilation and FP.

### 2.5 Absorptive capacity

The notion of AC, introduced initially in macroeconomics by Ref. [99], highlights a firm's capacity to effectively utilize, assimilate, and exploit external resources and knowledge for commercial purposes. The concept of AC is a crucial factor in facilitating a manufacturer's expansion. This can be accomplished by effectively implementing a distinct set of intra-organizational procedures and gradually modifying and reorganizing the firm's dynamic capabilities, as [56] suggested. It is proposed that organizational learning capabilities play a pivotal role in resolving customer-related issues [100]. [101] found that AC and learning capacity have a positive and significant effect on the innovation of SMEs.

### 2.6 Hypotheses development

#### 2.6.1 Artificial intelligence assimilation and firm performance

AI-enabled firms are equipped with processes and services transformation, which enhances their organizational performance [102]. IT assimilation promotes business value creation [78] and improves a firm's effectiveness and efficiency [103]. Numerous studies have examined and reported a positive link between IT investments and FP [12,104]. Based on sample data collected from the electronic sector in China [104], examined the influence of IT investment on FP and reported a positive impact. In the healthcare industry [105], discovered that IT-oriented services improve hospitals' performance.

Consequently, big data analytics have been reported to enhance performance positively [106]. In parallel [8], found that AI Chatbots improve SMEs' sustainable supply chain performance. It was reported that employing AI can enhance firms' performance by reducing lead times, revolutionizing customer service standards, and delivering personalized experiences [107]. Furthermore, adopting AI has been suggested to enhance business procedures and FP in terms of administrative and financial performance [74]. Based on data collected from e-commerce organizations [108], reported a positive relationship between AI technology and FP. Similarly [11], based on data obtained from supply chain managers in the United States, reported that AI assimilation is a positive determinant of firm performance. However [109], uncovered that the effect of AI capabilities on business performance is partially mediated.

The literature offers an insufficient explanation of why the link between AI assimilation and FP has not yet been examined in developing economies. Hence, this is an initial effort to explore this relationship from the perspective of Lebanese SMEs. In line with the above evidence, we posit that.

H1 : Artificial intelligence assimilation has a positive effect on firm performance.

#### 2.6.2 Artificial intelligence assimilation and customer agility

[11] emphasized that organizations worldwide use AI capabilities to improve customer agility. From this standpoint, Amazon and Netflix are utilizing AI-enabled technologies to make personalized suggestions to their customers and subscriber base worldwide and promote their agility toward their subscribers and customers [110]. It is reported that the integration of AI-enabled chatbots into the firm's operations improves the firm's agility and customer service performance [87].

It has been proven in the literature that digital technologies can facilitate the implementation of clear-cut methods to make firms more agile [51] by detecting consumers' opportunities and risks [53] and successfully reacting to them [111]. Further, modern facilities may significantly enhance CA [11,89]. IT has long been recognized as an essential tool for building and strengthening online customer relationships and improving CA [47] Such customer relationships may improve interactions between firms and customers while obtaining information for product design, testing, or review [51]. Additionally, AI improves customer forecasting intelligence that can be utilized to strengthen firm processes, enable customized offers and services, and predict and reduce customer attrition [112]. Moreover, AI is applicable in various interdisciplinary systems and boosts companies' ability to deliver satisfaction to their customers [113]. [114] indicated that AI significantly impacts the customer experience. Most of these studies were conducted in Western countries; in advancing the existing literature, we propose.

H2 : Artificial intelligence assimilation has a positive effect on customer agility.

#### 2.6.3 Customer agility and firm performance

CA enables firms to continually adapt to customer-oriented opportunities by responding proactively and launching new promotions and services/products to increase profitability, industry position, and competitive edge [54]. Previous research has also illustrated that it can forecast new products' achievement [52] and expedite the manner and speed in which organizations detect and take advantage of innovative possibilities [52] to create a novel product/service that enhances organizational performance [74]. It is easy for firms with greater CA to match customers' demands and needs with their products and services, significantly affecting customer satisfaction [96]. Specifically, organizations such as Amazon and Netflix utilize AI-powered personalized product recommendations, individualized marketing, and price optimization to boost CA and enhance sales [110,115]. Based on the above arguments, we posit that.

H3 : Customer agility has a positive effect on firm performance.

#### 2.6.4 Artificial intelligence assimilation and absorptive capacity

It is anticipated that firms can benefit from AI assimilation as a new knowledge source while using it as a tool for learning. This could impact the concept of AC, connecting with a firm's changing capability that companies' technology choices can have an impact. Indeed, it is surprising that empirical examination of the impact of AI assimilation on AC is relatively scarce in the extant literature.

Based on healthcare samples, prior studies have suggested that strong assimilation of technological trends, including considerable data-artificial intelligence (BDA-AI), can facilitate AC in healthcare processes [116] and boost healthcare organizations' innovative performance [117]. From this standpoint, the technological benefits of BDA-AI facilitate AC to deliver effective organizational operations [118]. It is argued that a strong AI assimilation can favorably impact AC in SMEs. Based on the above argument, we posit that.

H4 : Artificial intelligence assimilation has a positive impact on absorptive capacity.

#### 2.6.5 Absorptive capacity and firm performance

Numerous studies documented in the literature have reported a favorable impact of AC on business performance and innovation outcomes. For instance, the research conducted by Ref. [119] reported a link between AC and new product development. Similarly, several studies based on SME samples reported that AC increases FP [[120], [121], [122], [123]]. In addition, absorptive capacity, as noted, has been identified as crucial for the hotels' performance [124]. However, most studies were conducted in developed economies within the Western context. This limits the generalization of the relationship. Likewise [125], reported that absorptive capacity positively impacts firms’ performance by improving financial performance. In building the existing literature in the context of Lebanese SMEs, we posit that.

H5 : Absorptive capacity has a positive effect on firm performance.

#### 2.6.6 The mediating role of customer agility

To obtain information and data regarding customer-oriented opportunities enabling innovations and competitive actions, firms must establish customer-centric programs [53] to develop their ability to identify market opportunities and build the capacity to generate and leverage knowledge [126,127]. Based on these arguments, previous research has highlighted customer analytics as a key mechanism of the link between IT infrastructures and FP [54,128]. Further, IT infrastructure enables CA, which drives competitive activities [53]. The authors further argued that agility is a mechanism that gives rise to economic implications and offers an alternative perspective on how IT infrastructures indirectly improve business value.

CA enables businesses to restructure and optimize their business operations to obtain precision, cost-effectiveness, and speed in their organizational procedures. This capability is the bedrock for modern firms in handling instability and uncertainty [129]. Similarly, firms that promptly respond to customers' changing demands using their IT capabilities [53,130] will likely achieve cost-effectiveness [129]. In line, by adopting AI-powered personalization, firms are empowered to support their customers with customized experiences, significantly enhancing marketing efficiency and overall business profitability and performance [131].

Although the link between AI assimilation and FP has been examined [47], no consensus has been reached on whether CA would mediate this relationship in the context of developing economies. However [115], found CA to be a complementary partial mediator of the nexus between AI assimilation and FP. Building upon the above arguments and bridging gaps in the literature, this study examines CA as an underlying mechanism in Lebanese SMEs. Thus, we posit that.

H6 : Customer agility mediates the link between artificial intelligence and firm performance.

#### 2.6.7 The mediating role of absorptive capacity

Scholars have categorized AC as a dynamic capability [132]. Consequently, organizations with greater AC are better equipped to leverage knowledge-based resources, detect technological advancements, and adapt functional capabilities to enhance their market offerings [133], which may improve firm performance. Further, the degree of AC exhibited by an organization plays a crucial role in determining the effectiveness of its innovation [133]. According to Ref. [134], it is possible to integrate consumers' information into advanced technologies to generate more extensive customer’ profiles, improve insights, boost personalized offers, and increase customer experiences. Such actions allow organizations to enhance their offerings by acquiring cognitive technological capabilities, including AI capabilities for performance enhancements [135].

The ability to absorb external knowledge is seen as a crucial factor for business model adoption [136]. From this standpoint, SMEs with robust AC can quickly detect new opportunities and better use new technologies for operational efficiency. Furthermore, it has been reported that IT capabilities positively influence service and product innovation, and AC mediates this relationship [137].

Customer data can be obtained through firm-level AC, which can then be internalized and integrated into technologies (such as machine learning models) to establish more detailed customer profiles and experiences [134], which may improve firm performance. Based on the discussion and previous empirical evidence, we posit that.

H7 The link between AI assimilation and firm performance is mediated by absorptive capacity.

#### 2.6.8 The moderating role of organizational agility

As stated in the previous sections, the links between AI assimilation, CA, and FP are still far from being clearly understood in the extant literature, particularly in the context of developing economies. While the current research proposed hypotheses posited positive relationships among the constructs, dynamic capabilities theory can offer a better understanding of the relationships among the aforementioned constructs. The literature has extensively used dynamic capabilities theory to comprehend the relevance of an organization's strategic capabilities and their influence on competitive performance [62,126,127]). The dynamic capabilities theory suggests that firms can transform their current positions, operational capabilities, and resources, resulting in new positions and alternative paths to maximize the effectiveness of key assets [62]. Numerous studies have applied DC theory to comprehend IT-based capabilities and firm-level outcomes [11,64,138]. In line with DC theory, we suggest OA as a driving force that can either weaken or strengthen the relationships in this study's integrated theoretical model, which will be addressed in more detail below.

While IT competence allows an organization to enhance its performance, the extent to which this option is exercised may rely on how agile the firm is in a given sector. Strong OA can assist firms in maximizing the use of their existing information and capabilities, putting them in a better position to identify and capitalize on opportunities in a target market [90,139]. From this standpoint, in a volatile market characterized by uncertainties, a strong OA can help firms align IT capabilities with changing market requirements and demands and overcome constraints while enhancing their performance [[140], [141], [142], [143]].

Furthermore, during market turbulence, firms are subjected to changes in their business models and must adjust their business processes, even their fundamental operations, to stay ahead of their competition [144]. Due to this, firms may need to increase their investment in IT to enhance their operational efficiency and sustain a competitive edge [145]. Additionally, a more effective use of resources, particularly IT, would enhance FP [146]. These arguments suggest that a strong OA enhances the ability of SMEs to invest in continual adjustment of their market performance, which can be used to reduce market turbulence and improve resource efficiency, such as cost improvements (i.e., firm performance). High OA is anticipated to benefit SMEs in the current digital era of technological breakthroughs where firms can better sense and efficiently react to the constantly changing market environment. OA may impact the positive link between AI assimilation and AC by promoting firms to exploit new opportunities and reconfigure their knowledge skills and banks to match the changing business environment.

Furthermore, certain studies have suggested that IT capabilities may not constantly improve firm-level outcomes [129,147,148]), particularly if they do not align with business agility needs [149,150]. Thus, a high OA can allow SMEs to develop and reconfigure their resources, particularly AI assimilation, in response to unanticipated market shifts, enabling them to handle imminent threats and emerging opportunities. Hence, SMEs that exhibit evidence of OA can readily improve their operation niche, adapt rapidly to market uncertainty, fulfill the expectations of their customers, and generate new opportunities, which can collectively strengthen their overall performance. Thus, we posit the following.

H8 : Organizational agility moderates the positive link between AI assimilation and customer agility, such that the positive relationship is further enhanced at a higher level of organizational agility.

H9 : Organizational agility moderates the positive link between AI assimilation and firm performance, such that the positive relationship is weakened at a lower level of organizational agility.

H10 : Organizational agility moderates the positive link between AI assimilation and absorptive capacity, such that the positive relationship is further enhanced at a higher level of OA.

## 3 Methods and results

### 3.1 Research context

SMEs play a crucial role in the world economy. Specifically, they account for 90 % of businesses in Lebanon and serve as a source of employment for more than half of the nation's labor force [151]. However, 50 % of small businesses fail during their early stages due to insufficient capabilities, expertise, and resources [152,153]). Additionally, it has been suggested that AI will play a crucial role in how SMEs evolve and perform in the coming decades [30]. To this end, the Lebanese government is currently taking measures to support technological advancement in the nation, especially in the field of AI, and to support businesses [154]. This makes Lebanon an interesting research context for our study because it is crucial for SMEs to understand the impact of technologies on their businesses. Furthermore, it is essential to understand how and when (under what conditions) AI assimilation leads to FP in the SME context.

### 3.2 Sample and data collected

The present study utilizes a quantitative research approach, specifically employing a questionnaire survey to collect data and gain knowledge of the proposed relationships illustrated in the moderated multi-mediation model presented in Fig. 1. The survey participants of this study were middle and upper-level managers in charge of business information management of SMEs in Lebanon. Considering that these SMEs in our sample hardly publish documents, we acquired the primary data through on-site questionnaires. To ensure that the subjects are true representatives of the sample, two screening questions were asked: First, "Does your firm apply business analytics in your firm operations?" In which areas? Second, "Does your firm apply AI-based application systems in your firm operations?" Share some examples. Data was collected through self-report questionnaires in Beirut, Tripoli, Mount Lebanon, Saida and Zahle. These cities house the major concentration of SMEs in Lebanon. It was ensured that only necessary participants were targeted [155]. They include middle and upper-level managers of SMEs with established AI-equipped application systems who understand the requirements of their enterprises concerning both non-technical and technical capabilities related to AI. Unlike prior works (e.g., Refs. [[156], [157], [158]]), we employed a non-probabilistic sampling method. The rationale behind adopting this sampling technique was to obtain accurate and comprehensive information regarding companies that prioritize and use AI/IT-related technologies and capabilities. Subsequently, a sample of the selected firms, whose middle and upper-level managers demonstrated knowledge and expertise while responding to the screening questions, was randomly chosen and contacted before the survey distribution to explain the study's reasons. Data collection took place between September 2022 and February 2023. The selection process involved 604 SMEs in Lebanon, which collectively employ approximately 3200 employees. The sample size "417″ was determined through the widely accepted Slovin formula, which is a global standard for estimating sample size. This approach has recently been used on SMEs [125].

$$n=\frac{N}{1+N\left(e\right)ˆ2}$$

$$n=\frac{3200}{1+3200\;\left(0.05\right)ˆ2}$$

n=355

1099 questionnaires were distributed. A total of 465 responses were recovered, of which 48 incomplete responses were discarded, and 417 valid responses (well above 355) were retained for the data analysis. Thus, the study yielded a response rate of 37.94 %.

The demographic characteristics of the survey respondents are illustrated in Table 1. On gender, 218 (52.28 %) participants were males, while 199 (47.72 %) were females. Based on education, the majority of the respondents, 317 (76.10 %), had at least a bachelor's degree, implying that they had sufficient academic qualifications to rate the constructs of this study. The respondents were from various industries: food and beverages 166 (39.81 %), consulting 86 (20.62 %), transportation 72 (17.27 %); retail and wholesale trade 53 (17.27 %), and computer and software 40 (9.59 %). Based on firm age, 249 (59.71 %) of the participants were from firms that have been in business for less than 20 years, while 168 (40.29 %) were from firms that have been in business for over 20 years. In terms of firm size (i.e., number of employees), 61 (14.63 %) of the respondents were from firms with less than 20, while the majority 356 (85.37 %) were from firms with over 20 employees.

**Table 1:** Demographic details.

Demographic Information (N = 417)	Frequency	Percentage (%)
**Gender**		
Male	218	52.28
Female	199	47.72
**Education**		
High School	100	23.98
Bachelor	210	50.36
Masters	83	19.90
PhD	24	5.76
**Industry**		
Computer/Software	40	9.59
Consulting	86	20.62
Food and Beverages	166	39.81
Transportation	72	17.27
Retail and Wholesale Trade	53	12.71
**Firm Age (years)**		
Less than 20	249	59.71
21–40	126	30.22
41–60	40	9.59
Over 60	2	0.48
**Firm Size (Number of employees)**		
Less than 20	61	14.63
21–40	95	22.78
41–60	108	25.90
61–80	83	19.90
81 or more	70	16.79

### 3.3 Statistical techniques

In this study, descriptive statistics were analyzed using SPSS 27.0 to search for missing data and pertinent outliers and to determine whether the data collected was normally distributed. In addition to this, descriptive statistics were computed to gain an understanding of the frequency and percentage of the demographic information of the respondents.

This research utilized confirmatory factor analysis (CFA) with AMOS 24 as the statistical software to estimate the measurement model and explore the scale's relevant psychometric aspects.

Furthermore, we employed Haye's PROCESS macro (Model 4), suggested by Ref. [159], to examine the multi-mediation model. Finally, the moderated multi-mediation model was examined using PROCESS model 8, which was recommended by Ref. [160]. A Bootstrap with 5000 resamples and a 95 % confidence interval (CI) must not include zero for a significant indirect effect to be observed [32,161].

### 3.4 Common method bias (CMB)

To minimize the consequences of the common method bias, this research utilized procedural as well as statistical measures. In terms of the procedural measure, we made sure the questionnaire was clear and precise, provided the measurement items for each construct in separate parts, and chose experienced middle-level and upper-level managers as participants (almost 87 % of whom had more than five years of work experience), and made sure the respondents' anonymity was fully protected [162]. Due to the employed methods, participants could respond thoughtfully and honestly to the questions. As a result, we were able to acquire reliable information sources.

Additionally, we used exploratory factor analysis (EFA) for statistical measures to carry out Harman's single-factor test. The EFA results demonstrate that there were five different factors, each having an eigenvalue that is above 1. Together, they explained 67.22 % of the total variance. Only 38.45 % of the total variance could be explained by the first factor extracted. This is less than the recommended 50 % threshold, implying that CMB is not a significant concern in the present research. Further, it was also determined that there was no evidence of multicollinearity on the estimated coefficients by estimating the variance inflation factors (VIF) for each construct, and they were all found to be less than five [163,164].

### 3.5 Measurement model

Confirmatory factor analysis (CFA) was used to estimate the measurement model and psychometric validity of the present study constructs (AI assimilation, CA, OA, and FP). The results revealed that all fit measures (absolute ′fit, incremental fit, parsimonious fit, and the comparative fit index) were determined to be satisfactory via CFA analysis, as presented in Table 2. Particularly, this study evaluated absolute fit measures by analyzing GFI, AGFI, RMSEA, and RMR values. Indexes like AGFI (0.905) and GFI (0.929) have higher values than 0.90. RMR (0.032) and RMSEA (0.044) values were less than 0.08, demonstrating the measurement's model absolute fit.

**Table 2:** CFA (model fit summary).

Fit Measures	Criteria (Assessment)	Thresholds	Obtained Values	Remarks
Absolute Fit	CMIN/DF	≤3	324.149/157 = 2.065	[169]
	GFI	≥0.9	0.929	
	AGFI	≥0.9	0.905	
	RMR	≤0.08	0.032	
	RMSEA	≤0.08	0.044	[170]
Incremental Fit	CFI	≥0.9	0.975	CFI, NFI, TLI, RFI:
	NFI	≥0.9	0.954	[171,172]
	TLI	≥0.9	0.970	
	RFI	≥0.9	0.944	
Parsimonious Fit	PNFI	≥0.7	0.788	[173,174]
	PCFI	≥0.7	0.806	
	PGFI	≥0.7	0.701	

Indexes such as CFI, NFI, TLI, and RFI were used to estimate the measurement's model incremental fit; CFI (0.975), NFI (0.954), TLI (0.970), and RFI (0.944) were all above the recommended values, confirming the incremental fit of the measurement model. Furthermore, this research used two fit indices, PGFI and PNFI, to validate the parsimonious fit measures and circumvent the probable issues associated with absolute and incremental measurements. As illustrated in Table 2, the indices exceeded the recommended threshold of 0.7 and hence considered satisfactory.

The resulting CFA model fits the data extremely well, with acceptable factor loadings for each measurement item. The measurement model should be examined for the reliability of individual items (factor loadings), internal consistency, and validity (convergent and discriminant) [165]. The factor loadings of measurement items within each construct are within an acceptable range (0.673–0.912), as shown in Table 3 [166]. recommended that Cronbach's alpha and CR values for the variables should be higher than 0.7. As shown in Table 3, the Cronbach alpha values (ranging from 0.830 to 0.927) and the CR values (ranging from 0.840 to 0.934) were higher than the recommended thresholds. Further, each AVE was greater than 0.5 (ranging from 0.567 to 0.825), as displayed in Table 3, and the square roots of all AVEs were greater than the correlation of the surrounding constructs [167,168]. Therefore, the measurement model can be considered reliable and valid for further analyses.

**Table 3:** Measurement model assessment.

VN	M	SD	FL	Α	CR	AVE	MSV	AIA	CA	AC	OA	FP
**AIA**	4.216	0.813		0.927	0.934	0.825	0.427	**0.908**				
AIA1			0.912									
AIA2			0.906									
AIA3			0.907									
**CA**	4.006	0.716		0.916	0.916	0.731	0.359	0.555**	**0.855**			
CA1			0.863									
CA2			0.847									
CA3			0.868									
CA4			0.843									
**AC**	3.997	0.744		0.830	0.840	0.567	0.326	0.499**	0.512**	**0.753**		
AC1			0.673									
AC2			0.701									
AC3			0.789									
AC4			0.809									
**OA**	3.957	0.775		0.927	0.924	0.603	0.427	0.602**	0.525**	0.611**	**0.777**	
OA1			0.789									
OA2			0.820									
OA3			0.859									
OA4			0.736									
OA5			0.723									
OA6			0.707									
OA7			0.872									
OA8			0.740									
**FP**	3.941	0.733		0.903	0.905	0.656	0.238	0.451**	0.416**	0.404**	0.506**	**0.810**
FP1			0.787									
FP2			0.851									
FP3			0.839									
FP4			0.787									
FP5			0.783									

Note: VN= Variable Name; M = Mean; SD= Standard Deviation; FL=Factor Loading; α = Cronbach's Alpha; CR= Composite Reliability; MSV = Maximum Shared Variance; AIA = Artificial Intelligence Assimilation; CA= Customer Agility; AC = Absorptive Capacity; OA= Organizational Agility; FP= Firm Performance; ** = correlation significant at 0.01 level.

### 3.6 Test for parallel mediation model

Model 4 of PROCESS by Hayes (2013) was used to explore the parallel mediation model [161,175]. pointed out that researchers commonly employ parallel multiple mediation models in substantive fields to comprehend how a significant relationship can be mediated by two variables. Through the use of Haye's PROCESS model 4, hypotheses H1 to H7 were examined. The results of the parallel mediation model revealed that AI assimilation has a positive significant impact on CA (β = 0.489, p < 0.000, CI 95 % = 0.418, 0.560) in Table 4, Model 1. AI assimilation has a positive significant effect on AC (β = 0.286, p < 0.000, CI 95 % = 0.195, 0.377) in Table 4, Model 2. AI assimilation positively and significantly predicted FP (β = 0.227, p < 0.000, CI 95 % = 0.126, 0.327) in Table 4, Model 3. CA significantly and positively predicted FP (β = 0.204, p < 0.01, CI 95 % = 0.096, 0.311) in Table 4, Model 3. AC significantly and positively predicted FP (β = 0.204, p < 0.01, CI 95 % = 0.096, 0.311) in Table 4, Model 3, thus validating H1, H2, H3, H4, and H5.

**Table 4:** Results for mediation analysis.

Mediation Analysis: Customer agility and absorptive capacity mediated (partially) the link between artificial intelligence assimilation and firm performance (PROCESS: model 4, CI = 95 %) Bootstrap 95 % CI							
	**Β**	**SE**	**T**	**Sig**	**LLCI**	**ULCI**	**R**^**2**^
**Model 1**: mediator variable model Artificial Intelligence Assimilation	Outcome: **CA** 0.489 0.036 13.602			0.000	0.418	0.560	0.308
**Model 2**: mediator variable model Artificial Intelligence Assimilation	Outcome**: AC** 0.286 0.0466.174			0.000	0.195	0.377	0.243
**Model 3**	**FP**						
Artificial Intelligence Assimilation	0.227 0.051 4.423			0.000	0.126	0.327	0.256
CA	0.204 0.055 3.734			0.002	0.096	0.311	
AC	0.139 0.053 2.660			0.081	0.036	0.243	
**Bootstrap results for the indirect effects**							
(Indirect effect of AIA on FP via CA)	0.120	0.030			0.062	0.180	
(Indirect effect of AIA on FP via AC)	0.080	0.032			0.041	0.119	

Note: n = 417; Bootstrapped sample = 5000; Lower = Lower-level of confidence interval; Upper = Upper-level of confidence interval; Sig = Significance.

Furthermore, the bootstrapping method was used to perform mediation analysis. The bootstrap procedure has become the standard method for mediation analysis due to its simplicity and accuracy [160]. Using resamples of the given data to draw inferences and gain an understanding of the underlying population is one of the distinctive features of bootstrapping [176]. The current study used 5000 resamples to generate accurate results for the mediation model. According to the findings in Table 5, there is a significant positive indirect association between AI assimilation and FP through CA (β = 0.120, SE = 0.030, CI = 0.062, 0.180), and there is a significant positive indirect association between AI assimilation and FP through AC (β = 0.080, SE = 0.032, CI = 0.041, 0.119) in Table 4. Thus, H6 and H7 were supported with a confidence interval that did not include zero, unveiling CA and AC as parallel mediators.

**Table 5:** Moderated Multi-Mediation: Organization Agility moderated the direct and indirect relationship between Artificial Intelligence Assimilation and Firm Performance (model 8, 95 % CI).

Bootstrap 95 % CI							
	B	SE	T	P	LLCI	LLCI	R^2^
**Model 1**: mediator variable model	**Outcome: Customer Agility**						
Artificial Intelligence Assimilation	0.398	0.029	9.552	0.000	0.362	0.449	0.238
Organizational Agility	0.526	0.025	11.634	0.000	0.537	0.707	
Artificial Intelligence Assimilation X Organizational Agility (interaction)	0.071	0.016	3.002	0.000	0.174	0.232	
Co: Age	0.089	0.006	0.404	0.631	−0.025	0.087	
Co: Education	0.169	0.014	1.628	0.042	0.089	0.103	
Co: Firm Size	0.072	0.016	0.416	0.529	−0.019	0.099	
**The conditional direct effect of Artificial Intelligence Assimilation on Customer Agility**							
Organizational Agility (-1SD)	0.174	0.074	7.231	0.022	0.050	0.221	
Organizational Agility (+1SD)	0.283	0.101	9.441	0.001	0.202	0.386	
**Model 2**: mediator variable model	**Absorptive Capacity**						
Artificial Intelligence Assimilation	0.198	0.057	4.019	0.007	0.114	0.299	0.267
Organizational Agility	0.200	0.054	4.088	0.000	0.119	0.307	
Artificial Intelligence Assimilation X Organizational Agility (interaction)	0.009	0.002	0.535	0.076	−0.067	0.109	
Co: Age	0.076	0.013	0.306	0.422	−0.014	0.066	
Co: Education	0.044	0.049	0.295	0.337	−0.007	0.082	
Co: Firm Size	0.068	0.011	0.304	0.449	−0.010	0.074	
**Model 3:** dependent variable model **Dependent: Firm Performance**							
AI Assimilation	0.257	0.034	4.439	0.001	0.143	0.254	0.206
Customer Agility	0.205	0.044	4.011	0.000	0.176	0.298	
Absorptive Capacity	0.124	0.60	2.452	0.046	0.021	0.288	
Organizational Agility	0.406	0.046	8.001	0.000	0.335	0.499	
Artificial Intelligence Assimilation X Organizational Agility (interaction)	0.069	0.028	2.774	0.001	0.107	0.296	
Co: Age	0.033	0.004	0.306	0.593	−0.004	0.081	
Co: Education	0.039	0.007	0.418	0.662	−0.014	0.091	
Co: Firm Size	0.41	0.011	0.863	0.399	−0.015	0.099	
**The conditional direct effect of AI Assimilation on Firm Performance**							
Organizational Agility (-1SD)	0.251	0.072	2.953	0.001	0.202	0.438	
Organizational Agility (+1SD)	0.423	0.063	5.331	0.001	0.229	0.404	

Note: n = 417; Bootstrap resample = 5000; LLCI = Lower-level of confidence interval; ULCI= Upper-level of confidence interval; Co=Control variable.

### 3.7 Moderation mediation model

To examine the moderating role of OA on the link between AI assimilation and CA in H8, the link between AI assimilation and FP in H9, and AI assimilation and AC in H10, Model 8 of PROCESS was adopted. Simultaneously, education, age, and firm size were added as covariates. Table 5 presents the moderated multi-mediation model results.

**Fig. 2:**
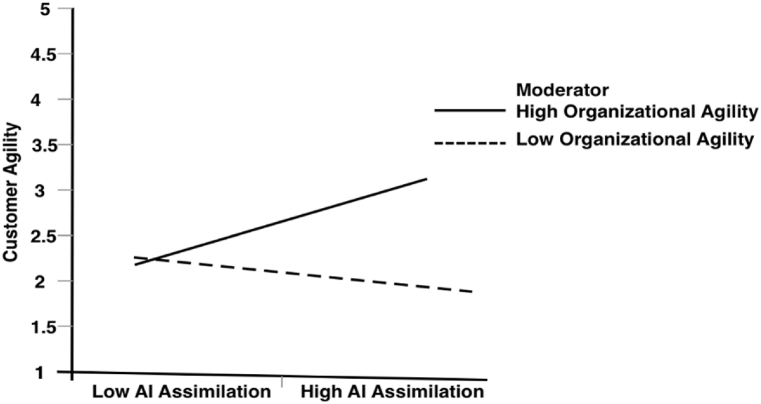
The moderating role of OA in the link between AI assimilation and CA (low OA = −1SD and high OA = +1S

It was revealed by the results that the effect of the interaction term between AI assimilation and OA on CA was significant (β = 0.071, p < 0.000). This validates H8, revealing that OA moderates the positive relationships between AI assimilation and CA. This interaction's significant effect was examined further using simple slope analysis. We plotted the interaction at +1 and −1 SD from the mean of OA (illustrated in Fig. 2). A simple slope was built to examine the strength of the link between AI assimilation and CA at high and low levels of OA. The results of the conditional direct effect indicated that the strength of the positive relationship was stronger for SMEs with high OA (β = 0.283, p=<0.001) compared with low OA (β = −0.174, p=<0.05), hence further supporting H8.

**Fig. 3:**
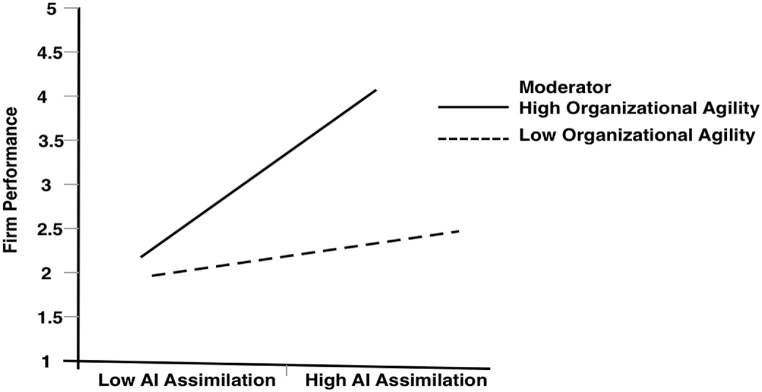
The moderating role of OA in the link between AI assimilation and FP (low OA = −1SD and high OA = +1SD).

It was also discovered that the interaction effect between AI assimilation and OA on FP was significant (β = 0.069, p=<0.001), implying that OA moderates the positive link between AI assimilation and firm performance. Similar to H8, the interactions were plotted at +1 and −1 SD from the mean of OA (illustrated in Fig. 3). We then built a simple slope analysis to explore the strength of the positive link between AI assimilation and FP at high and low levels of OA. The conditional direct effect results showed that the strength of the positive relationship was weakened for SMEs with low OA (β = 0.251, p < 0.001) and high for SMEs with high OA (β = 0.423, p < 0.001), hence offering validation for H9.

The obtained results indicated that the interaction effect between AI assimilation and AC on FP was insignificant (β = 0.069, p=>0.05), revealing that OA did not moderate the positive link between AI and AC.

## 4 Discussion

The current study explored the direct impacts of AI assimilation on CA, AC, and FP. The research also investigated the mediating roles of CA and AC on the link between AI assimilation and FP and the moderating role of OA in the relationships above.

The present research findings showed that AI assimilation had a significant positive effect on FP, validating (H1). This result is in line with the findings of [47,177,178] that AI assimilation improves FP. The discovery of a positive relationship between AI assimilation and FP in a developing economy in the context of SMEs in Lebanon highlights the novelty of our findings, showing that this particular relationship extends beyond the traditional confines of the Western context.

The research findings validated (H2), indicating that AI assimilation was a positive determinant of customer agility. Our results support the conclusions of [47,89] regarding the importance of IT tools in establishing CA and suggest that SMEs should leverage AI to enhance their processes, capabilities, and customer adaptability and thus, elevate their overall performance.

The third hypothesis (H3) was also supported. In this regard, CA was revealed as a positive determinant of FP. This finding substantiates the conclusion of the study of [52] concerning the positive impact of CA on organizations' product support and highlights the necessity of promptly addressing customers' concerns and innovating support services through cultivating and enhancing CA within the firm's structure and processes.

AI assimilation was also affirmed as a positive determinant of AC, offering validation for (H4). This result implies that AI assimilation enables firms to better understand and interpret market opportunities, leading to the acquisition of new insights and reinforcing their sensing capabilities.

Likewise, the fifth hypothesis (H5) was supported. AC was unveiled as a positive predictor of FP in the context of Lebanese SMEs. This result aligns with numerous prior studies such as those of [120,121], and [179]. Our study provides an empirical demonstration of the continuous alignment of this pattern of results, suggesting that AC improves a firm's ability to transform and integrate externally obtained information within the firm while enhancing its performance.

Our research goes beyond the conclusions in the previous literature about the roles of CA and AC and proposes them as parallel mediators in the relationship between AI assimilation and firm performance. The hypothesized parallel mediation was supported through the validation of (H6) and (H7). First, it was revealed that CA partially mediated the link between AI assimilation and FP, which empirically verifies previous related research [53,54] and aligns with the findings of [47]. Similarly, AC was found to play a partial intermediary role in the link between AI assimilation and firm performance. Our findings suggest that firms that possess the AC and CA feature the ability to effectively utilize and streamline externally obtained knowledge into their firm routines and foster adaptability and responsiveness to dynamic market demands. Moreover, they acquire the capability of assimilating such knowledge into their processes and operations, ultimately leading to reaping the benefits of advanced technologies for enhanced firm performance.

Remarkably, the findings supported (H8), indicating that OA moderated the positive link between AI assimilation and CA in that the strength of the positive relationship is higher for SMEs with high OA. Similarly, (H9) was validated, revealing that OA moderated the positive link between AI assimilation and firm performance, such that the strength of the positive relationship is weakened for SMEs with low OA. These findings indicate that agile firms are enabled to actively pursue quick responses driven by AI technologies to develop their capabilities to react swiftly and cost-effectively to unanticipated market changes in turbulent market environments. Furthermore, SMEs can optimize CA utilization within the firm processes by comprehending and strengthening OA. These findings extend our understanding beyond earlier scholarly literature conclusions on the impactful role of OA (e.g., Refs. [47,55]). In light of these results, this research proposes critical insights into how SMEs can attain competitive advantages, optimize AI utilization, minimize inefficiencies, and boost performance by leveraging the integrative relationships of AI and CA with OA and that of AI and FP with OA. Finally, (H10) was not supported, implying that OA does not moderate the link between AI assimilation and AC. Thus, OA cannot be assumed to be a universal moderator in this context.

## 5 Implications

### 5.1 Theoretical implications

Through advancing a moderated multi-mediation model, the current research offers several crucial contributions to the theorization and comprehension of AI assimilation by linking it with three organizational-level outcomes, particularly in the context of SMEs in Lebanon. To the best of our knowledge, this is the first study that uses the dynamic capabilities theory to examine the effect of AI assimilation on FP, in addition to the multi-mediating roles of CA and AC and the moderating role of OA on the relationships in a developing economy. Consequently, this constitutes an essential addition to the emerging knowledge on AI assimilation in the existing literature [47,102,107].

By offering empirical evidence on the crucial role of AI assimilation in enhancing FP in the SME context, particularly in a developing economy, we fill the void in the literature stream that is marked by a paucity of empirical evidence resulting from the limitations in data availability, particularly in the business sector [24]. Thus, in line with the results obtained by Refs. [10,47], this study expands our understanding of AI enablers and strengthens the notion that firms using AI-related technologies will only be able to establish and obtain business value if they are appropriately integrated into end-to-end firm processes.

As [133] pointed out, a comprehensive understanding of the relationship between AI assimilation and AC is still lacking in the existing literature. In response, by theorizing and providing empirical evidence that AI assimilation is a crucial determinant of AC, this study offers new empirical evidence that is lacking in the existing body of literature and extends AI assimilation and AC literature. Furthermore, the current research demonstrates how AI assimilation enables CA and AC and provides essential knowledge on how they enhance firm performance. These relationships have not been empirically proven in the context of SMEs in Lebanon, thereby demonstrating that they are not restricted solely to advanced economies.

The assimilation of AI is a unique and essential capability to enhance performance-related outcomes that scholars must constantly explore [19,47,180]. While AI assimilation drives FP [47], less is known about how this relationship occurs. Thus, the present study responds to the recent calls by several scholars [19,47,180]. This is accomplished by drawing on dynamic capability theory and identifying two crucial underlying mechanisms regarding how AI drives firm performance. Specifically, this study offers new evidence and reveals CA and AC through which AI assimilation improves FP in the SME context. Our study demonstrates how these mechanisms contribute and are involved in the process of improving the performance of SMEs, beginning with the use of AI-related technologies through CA and AC to achieve improved firm performance.

Finally, despite the importance of OA in improving firm-level outcomes (e.g., Ref. [47]), OA as an organizational condition remained unresearched. A crucial finding that is not present in the extant literature is that this study offers evidence on how OA moderates the relationship between AI assimilation and CA and between AI assimilation and FP. Instead of examining OA as a predictor [181] or as a mediator [47], this study took a different approach and examined OA as a moderator. Thus, a novel approach that exceeds the direct effect is deployed to reflect the complexities of realities. By offering empirical evidence that OA moderates the relationships in our integrated theoretical model, this study goes beyond the initial research of [47] on how AI assimilation translates into firm performance. Specifically, it reveals under what conditions the relationships between AI assimilation, CA, AC, and FP are either weakened or strengthened. Hence, it constitutes a major contribution to the Dynamic Capability View theory.

### 5.2 Managerial implications

The findings offer important practical implications for managers and SMEs seeking to invest in AI-powered business value solutions. Because of the scarcity of resources and capabilities of SMEs due to their size, they frequently encounter difficulties in the implementation of advanced technologies [138]. This study provides managers with recommendations for maximizing the utilization of AI. Accordingly, firms' managers can only develop and realize the full value of FP if they invest in AI assimilation across end-to-end firm processes and supplementary capabilities, including CA and OA.

The findings of this study can serve as a point of reference for managers and prompt them to give sufficient attention to how AI assimilation is developed. Managers should consider the necessary resources needed for AI assimilation to improve their capabilities. Resources of this kind consist of organizational IT infrastructure, a firm's digital culture, and employees' technological literacy, among others. Recognizing the merits of these and other categories of resources can significantly influence the capability of AI assimilation. As digital transformation in organizations is still in its early stages, especially in developing economies, AI assimilation can be hindered by insufficient employees with AI expertise to enable its adoption and integration into various firms' practices. Thus, firms must train their employees in IT assimilation-related skills.

The findings of the current research indicated that AI assimilation has a positive effect on CA. This finding should motivate the management of SMEs to gain an enhanced knowledge of how AI technologies can be used to support CA, particularly in complex and turbulent environments. In the modern world faced with unprecedented disruptions, AI assimilation can be markedly crucial for SMEs as they can use AI tools to gain more value for the customers and the firms.

The findings of this study also reveal the indirect role of CA in AI assimilation to firm performance. It shows that CA and AC complement the AI assimilation capability to achieve firm performance. Hence, the management of SMEs should pay sufficient attention to customers' needs to reap the benefits of AI assimilation for high firm performance. SMEs should strive to attain a higher degree of AC to integrate externally obtained knowledge into existing routines and expertise to implement novel resource configurations to benefit from market opportunities. This can enable the firm to create services and products that align with the ever-evolving business environment, thus promoting their performance.

Furthermore, the moderation results of OA can aid SME managers in comprehending the level of agility within their firm. This, in turn, emphasizes the impact of AI assimilation on both CA and overall business performance, highlighting the necessity for prompt actions to enhance SMEs' agility.

Lastly, our findings suggest that the management of SMEs should consider CA and AC as important complementary and OA as a crucial organizational factor when building central capabilities for business value creation and performance enhancements. This can be achieved by employing required strategies that support agility practices.

## 6 Conclusion, limitations, and future research

### 6.1 Conclusion

This article contributes to emerging studies on AI at the organization level by examining the effect of AI assimilation on FP, which so far has received limited attention, particularly in the context of developing economies, which remains under-researched, calling into question the generalizability of the findings and theories in this research area. Our study is an initial effort to address this issue. This study also offers insights into the mediating role of CA and AC on the link between AI assimilation and FP. We found that OA moderates the link between AI assimilation and CA and AI assimilation and FP in the SME context.

### 6.2 Limitations and directions for future research

This article has some limitations that future studies should take note of. First, the sample obtained is limited to SMEs in Lebanon because of cultural and technological disparities between nations; the conclusion of the present research may not be generalizable to other nations. Hence, future research should collect data from multi-cultural nations to boost the generalizability of the findings. Second, a complimentary qualitative or longitudinal study that gives more information about the causal relationship between AI assimilation and FP via CA could enhance our understanding of the topic. Third, several contextual constructs, such as uncertainty, structure, and culture, could exist between AI assimilation and FP and moderate this relationship. Finally, research regarding AI technologies in obtaining business value is still very scarce in developing economies; more empirical studies are required to broaden our understanding.

## Funding statement

The research received no specific grant from funding agencies in the public, commercial, or not-for-profit sectors.

## Data availability statement

Data will be made available on request.

## Ethics declarations

This study was reviewed and approved by the ethical committee at Cyprus International University.

## CRediT authorship contribution statement

**Mohamad Deeb Abdul Wahab:** Writing – review & editing, Writing – original draft, Validation, Software, Project administration, Methodology, Investigation, Formal analysis, Data curation, Conceptualization. **Mehrshad Radmehr:** Writing – original draft, Validation, Supervision, Methodology, Formal analysis, Data curation, Conceptualization.

## Declaration of competing interest

The authors declare that they have no known competing financial interests or personal relationships that could have appeared to influence the work reported in this paper.

## Figures and Tables

**Table:** 

	AI Assimilation	[182]	Strongly Disagree	Disagree	Neutral	Agree	Strongly Agree
1	AI tools are used across all our business units.						
2	AI tools are used for decision-making across all our business units.						
3	AI tools are used to support the development of new products and services.						
	**Customer Agility**	[53]	Strongly Disagree	Disagree	Neutral	Agree	Strongly Agree
1	We respond rapidly if something important happens with regard to our customers.						
2	We quickly implement our planned activities with regard to customers.						
3	We quickly react to fundamental changes with regard to our customers.						
4	When we identify a new customer need, we are quick to respond to it						
	**Absorptive Capacity**	[121]	Strongly Disagree	Disagree	Neutral	Agree	Strongly Agree
1	The search for relevant information concerning our industry is an everyday business in our company.						
2	Our management demands periodic cross-departmental meetings to discuss new developments, problems, and achievements.						
3	In our company there is a quick information flow, e.g., if a business unit obtains important information, it communicates this information promptly to all other business units or departments.						
4	Our company considers technologies and adapts them according to new knowledge.						
	**Organizational Agility**	[140]	Strongly Disagree	Disagree	Neutral	Agree	Strongly Agree
1	The firm responds to changes in aggregate consumer demand.						
2	The firm customizes a product or service to suit an individual customer.						
3	The firm reacts to new product or service launches by competitors.						
4	The firm introduces new pricing schedules in response to changes in competitors' prices.						
5	The firm expands into new regional or international markets.						
6	The firm Changes (i.e., expand or reduce) the variety of products/services available for sale.						
7	The firm adopts new technologies to produce better, faster, and cheaper products and services.						
8	The firm switches suppliers to avail of lower costs, better quality, or improved delivery times						
	**Firm Performance (**To what extent do the following statements reflect the current situation in your firm in the last 24 months?)	[183]	Not at all	Very low	Low	High	Very High
1	We are more profitable than our competitors						
2	Our sales growth exceeds that of our competitors						
3	Our revenue growth exceeds that of our competitors						
4	Our market share growth exceeds that of our competitors						
5	Overall, our performance is better than our competitors						

## Appendix

The impact of AI assimilation on firm performance in small and medium-sized enterprises: A moderated multi-mediation model.

### Questionnaire Survey

Please take a few moments to complete this questionnaire, which aims to explore the influence of AI assimilation on the performance of Small and Medium Enterprises (SMEs). Your responses will be treated confidentially, and the aggregated data will be used for research purposes only. Your participation is greatly appreciated.

Demographic Information.

Gender
  1. Male
  2. Female
Education
  1. High school
  2. Bachelors
  3. Masters
  4. Ph.D.
Industry
  1. Computers/Software
  2. Consulting
  3. Food and beverages
  4. Transportation
  5. Retail and wholesale trade
Firm Age (In years)
  Less than 20
  21–40
  41–60
  Over 60
Firm Size (Number of Employees)
  Less than 20 years
  21–40
  41–60
  61–80
  81 or more

## Footnotes

1 Several studies showed that FP and stability are impacted by other factors at the firm and country level (e.g. [[33], [34], [35], [36], [37], [38]].
